# Efficacy of Stellate Ganglion Block in Treating Long-Term COVID-19-Related Olfactory and Gustatory Dysfunction: A Case Series

**DOI:** 10.7759/cureus.40929

**Published:** 2023-06-25

**Authors:** Arun Kalava, Steven A Benyahia, Ramon Tico Calzada, Christine M Staat

**Affiliations:** 1 Anesthesiology, University of Central Florida College of Medicine, Orlando, USA; 2 Medical School, University of South Florida (USF) Health, Tampa, USA; 3 General Medicine, Universidad de Ciencias Medicas Andrés Vesalio Guzmán (UCIMED) Escuela de Medicina, San Jose, CRI; 4 Anesthesiology, University of South Florida (USF) Health, Tampa, USA

**Keywords:** ageusia, long-covid-19, covid-19 long term outcomes, loss of taste, loss of smell, stellate ganglion block (sgb)

## Abstract

Olfactory and gustatory dysfunction, including anosmia, parosmia, ageusia, and dysgeusia, are common long-term symptoms of coronavirus disease 2019 (COVID-19) infection. These symptoms can have a severe impact on quality of life of a patient, including psychological well-being. Stellate ganglion block (SGB) has recently been explored as a potential therapeutic intervention for these symptoms. In this case series, we present six patients with long-term COVID-19 symptoms and we detail how their symptoms evolved after an SGB. All SGB were performed under ultrasound guidance by the same physician. Patients had a right SGB during the initial visit, followed by a left SGB at a subsequent visit. All but one patient reported improvements in olfaction and gustation after the SGB. Our findings suggest that SGB may be a promising therapeutic intervention for patients with olfactory and gustatory dysfunction related to long-term COVID-19 symptoms. Further research is needed to confirm these findings and to explore the optimal treatment protocol.

## Introduction

Long-lasting impairment of smell and taste, encompassing anosmia, parosmia, ageusia, and dysgeusia, is frequently observed in individuals who have experienced coronavirus disease 2019 (COVID-19) [1]. These symptoms can severely impact patients' quality of life, affecting their ability to enjoy food and detect potentially harmful odors [2]. Despite the growing number of patients affected by these symptoms, there is limited evidence of effective treatments for olfactory and gustatory dysfunction in long-term COVID-19 symptoms [3].

Stellate ganglion block (SGB) has recently been explored as a potential therapeutic intervention for olfactory and gustatory dysfunction in patients with long-term COVID-19 symptoms [4]. SGB involves the administration of a local anesthetic, such as ropivacaine, to the stellate ganglion, a collection of sympathetic nerves located anterior to the neck of the first rib. This procedure has been used to treat various pain conditions and has shown promise in alleviating post-viral anosmia and ageusia [4,5].

This case series aims to add to the growing body of evidence on the efficacy of SGB in treating olfactory and gustatory dysfunction in patients with long-term COVID-19 symptoms. We present six patients with persistent olfactory and gustatory dysfunction who underwent a series of SGB injections and 83% of the patients experienced significant improvements in their symptoms. This case series highlights the potential of SGB as a therapeutic intervention for long-term COVID-19-related olfactory and gustatory dysfunction and calls for further research to confirm these findings and explore the optimal treatment protocol.

## Case presentation

Procedural technique

Ultrasound-Guided Stellate Ganglion Block

After obtaining informed consent, the patients were brought to the procedure room and placed in Fowler's position. Blood pressure, heart rate, oxygen saturation and visual and verbal monitoring were established. A time out was performed. The area was prepped with 2% chlorhexidine gluconate and 70% isopropyl alcohol and draped in a sterile fashion. Next, after skin infiltration with 3 mL of 1% lidocaine, a linear ultrasound probe was placed in the anterior part of the neck to identify the stellate ganglion, which is located between the carotid artery and longus coli muscle at the level of the C6 transverse process in 80% of patients. A 10cm 22 Gauge nerve block needle (Havel's Inc, Cincinnati, OH, USA) was inserted using an in-plane approach, next to the stellate ganglion. After negative aspiration of blood, 7 mL of 0.5% bupivacaine or ropivacaine were slowly injected with alternating periods of aspiration that were negative for blood. In Video 1 there is an ultrasound capture detailing anatomical landmarks and showing the injection and spread of local anesthetic into the stellate ganglion.

**Video 1:** Ultrasound-guided stellate ganglion block This video details an ultrasound-guided stellate ganglion block, displaying the main anatomical structures and the spread of local anesthetic in the area of the stellate ganglion. Permission for use granted by the American Society for Post Surgical Pain

The patients were monitored up to 15 minutes after the procedure and were called for a follow-up the next day to assess symptom severity or changes if any. Additional follow-up times varied between one month and four months following the second SGB, with the specific timing varying for each patient.

This report presents six patients who experienced persistent anosmia, parosmia, ageusia, and dysgeusia after recovering from COVID-19 and underwent SGB injections to manage these symptoms. It is important to note that the improvement assessment is based on self-reported patient feedback.

Case 1

A 57-year-old male, weighing 95.0 kg, reported prolonged anosmia and ageusia persisting for five months following recovery from COVID-19 in August 2022. The patient reported that his sense of smell has been more affected than his taste. He also noted neuropathy in his left thumb, predominantly characterized by numbness. However, he has no complaints of neck pain. His past medical history reveals that he is pre-diabetic. He currently does not take any medications and has no known allergies. The first SGB, administered on the right side, resulted in an 80% improvement in taste and a 25% return of smell. Subsequent left-sided SGB led to a slight enhancement in both senses. At the three-month follow-up, the patient reported a greater improvement in taste compared to smell.

Case 2

A 48-year-old female weighing 63.5 kg reportedly contracted COVID-19 four times, with dysgeusia and anosmia occurring after her most impactful episode in November 2020. She reported experiencing dysgeusia and anosmia approximately 50% of the time, with altered sensations during episodes when taste and smell are restored. 

The patient has no significant medical history other than her COVID-19 infections. She does not take any medications and has no known allergies. The patient has explored acupuncture, aromatherapy, and hypnotherapy to relieve her symptoms, but these interventions have not led to any noticeable improvements.

The patient first received a right-sided SGB, and she self-reported a substantial 90% improvement in taste and a modest 10% return of smell based on her previous experiences. After the second left-sided SGB, she experienced a complete return of taste and a 30% return of smell. 

Case 3

A 36-year-old female weighing 56.7 kg, with a history of rheumatoid arthritis, contracted COVID-19 in August 2021. Since her recovery, she has been grappling with long-term symptoms such as anosmia, parosmia, ageusia and dysgeusia. She reported a total loss of smell for two weeks after a COVID-19 infection. Upon its return, she found that most smells had become unpleasant, faint, and seemed chemical in nature; her taste was altered as well. The patient has previously attempted smell retraining therapy which she states has led to minor improvements in her sense of smell. Additionally, the patient had undergone an MRI of the brain prior to receiving therapy, and no significant results were reported.

After her first right-sided SGB treatment, she reported a 70% return of smell and a 15% improvement in taste after the first right-sided SGB. The subsequent left-sided SGB resulted in an 80% return of smell and a 30% improvement in taste. At her one-month follow-up, the patient reported a cumulative 85% improvement in smell and a 50% improvement in taste, with continuous daily progress. At the three-month follow-up, she says that her smell has remained at 85% improvement, but she has experienced an 80% improvement in taste.

Case 4

A 20-year-old female weighing 52.2 kg, with a history of migraine headaches, with no long term medication use, contracted COVID-19 in December 2020. The patient initially experienced ageusia that lasted for six months; upon return of taste the patient had complaints of dysgeusia that lasted for over 1.5 years. The first SGB on the right side resulted in a 50% improvement in her symptoms. The second left-sided SGB led to an additional 30% improvement of her symptoms. After the initial follow-up, patient could not be reached for further follow-ups.

Case 5

A 55-year-old female, 62.1 kg, with dyslipidemia treated with atorvastatin, had anosmia and ageusia for 1.5 years following a week of respiratory symptoms due to COVID-19. An otolaryngologist had ruled out any anatomical abnormalities. She tried smell training without any improvement. Despite experiencing no initial improvement 24 hours after the right-sided SGB, a subsequent left-sided SGB was performed without complications. At the three-week follow-up period the patient says she has not noticed any improvement in her sense of smell or taste.

Case 6

A 55-year-old male, 95.0 kg, with a medical history of hypertension managed with losartan and depression managed with citalopram, reported anosmia and ageusia for 1.5 years after a COVID-19 infection. Antihistamines and consultations with an otolaryngologist were not helpful. After a right-sided SGB, the patient reported 80% improvement in smell, with a less significant improvement in taste. One week later the patient underwent a left-sided SGB. After the left-sided SGB the patient could not be reached for further follow-ups.

## Discussion

This case series showcases six patients who had SGB for the management of prolonged olfactory and gustatory dysfunction associated with long-term COVID-19. Five out of six patients demonstrated varying degrees of taste and smell perception improvement following the SGB. These findings offer preliminary evidence supporting the potential effectiveness of SGB as a therapeutic strategy for this cohort of patients, thereby paving the way for more rigorous investigations in the future [6-7].

Post-viral olfactory and gustatory dysfunction are increasingly recognized sequelae of COVID-19, with significant implications on quality of life. It is hypothesized that these symptoms could be due to the bilateral cervical sympathetic chain being affected during these viral infections, and that dysautonomia could play an important role in the pathophysiology of long COVID. Dysautonomia is not specific to COVID-19 infection, it has been reported with other viral illnesses such as HIV, Epstein-Barr virus, mumps, and coxsackie B [8]. Other conditions that are associated with systemic inflammation such as obesity, diabetes, and alcoholism can also cause dysautonomia. The autonomic nervous system plays a role in mediating the immune system to combat infections through the release of catecholamines [9]. It is believed that these conditions can cause the autonomic nervous system to enter a vicious cycle that perpetuates a proinflammatory state in the body that can end up causing dysautonomia, among other conditions seen in more severe COVID-19 infections [5]. There is also impaired cerebral blood flow reported in patients with dysautonomia, which can also affect the areas of the brain that mediate these sensory functions [10].

Due to this pathophysiology, SGB-mediated sympathectomy has been used as a therapeutic option in this patient population. It is hypothesized that SGB attenuates sympathetic hyperresponsiveness, and this causes a recalibration of the autonomic nervous system towards its pre-COVID state. Another mechanism described in other studies is that after SGB, there is increased cerebral blood flow [1]. This quickly improves perfusion of the cortical areas and peripheral receptors responsible for taste and smell and could be a reason that explains why some patients have such quick symptomatic relief hours after their first SGB [5]. The combination of these two mechanisms could explain why the duration of the resolution of symptoms outlasts the duration of the local anesthetic. Long COVID is a relatively new disease and we learn more about it every day. More studies are needed to identify the specific mechanism of why SGB causes resolution of symptoms related to taste and smell.

There is limited published literature on the use of SGB for long-term COVID-19 olfactory and gustatory dysfunction, and our case series adds to this growing body of evidence [1,3-8,10-11]. However, we acknowledge that our report carries the inherent limitations of a case series, including the need for a control group, the relatively small sample size, and the reliance on self-reported outcomes. Future randomized controlled trials are necessary to provide more definitive evidence of the efficacy and safety of this procedure in the context of long-term COVID-19 sensory dysfunction. Such trials should also consider more objective olfactory and gustatory function measurements, such as psychophysical testing, to corroborate patient-reported outcomes.

Considering the potential side effects and complications associated with SGB is noteworthy, as observed in our case series. These include temporary ptosis, conjunctival erythema, dysphonia, dysphagia, and hypopharyngeal anesthesia, among others. While the symptoms were transient and well-tolerated in our patients, it is essential for practitioners to counsel patients about these potential risks before performing the procedure.

It is also important to investigate the optimal timing and frequency of SGB for maximum therapeutic benefit. In our series, patients received one SGB on each side, but the time between procedures varied between patients. The variable times of doing contralateral SGB was governed by patient's ability to return for a subsequent visit, physician availability and distance between the physician's office and patient's residence. The timing of SGB administration in the course of disease progression may also influence outcomes, a facet not explored in this case series. These factors should be addressed in future research to optimize the therapeutic protocol.

## Conclusions

This study demonstrated promising results for SGB as a potential intervention for persistent olfactory and gustatory dysfunction following COVID-19. Among the six patients treated, five experienced considerable restoration of their taste and smell capacities. The self-reported patient improvement varied, from moderate to nearly full recovery of these senses, with an interesting trend of taste recuperation often preceding smell. Most of the positive outcomes were observed after bilateral SGB administration. However, the treatment did not lead to observable improvements in one out of six cases. While our findings highlight SGB as a possible remedy for the sensory dysfunctions associated with post-COVID-19 symptoms, they also underscore the need for more comprehensive research. Future studies must involve larger sample sizes and controlled experimental conditions to substantiate and broaden our understanding of this treatment's efficacy.
